# Is immediate weight bearing safe for periprosthetic distal femur fractures treated by locked plating? A feasibility study in 52 consecutive patients

**DOI:** 10.1186/s13037-016-0114-9

**Published:** 2016-12-07

**Authors:** Wade R. Smith, Jason W. Stoneback, Steven J. Morgan, Philip F. Stahel

**Affiliations:** 1 Mountain Orthopaedic Trauma Surgeons (MOTUS), Swedish Medical Center, Englewood, CO USA; 2 Department of Orthopaedics, University of Colorado School of Medicine, Aurora, CO USA; 3 Department of Orthopaedics, Denver Health Medical Center, 777 Bannock St, Denver, CO 80204 USA

## Abstract

**Background:**

Periprosthetic distal femur fractures associated with total knee replacement are increasing in incidence. We hypothesized that a standardized management protocol would result in few implant failures and a low rate of postoperative complications.

**Methods:**

Retrospective observational cohort study at an urban level 1 trauma center and academic level 2 trauma center. Consecutive patients with periprosthetic distal femur fractures and stable total knee arthroplasty were included between January 1, 2011 and December 31, 2014. Patients were managed by a standardized protocol of co-management by a hospitalist service, fracture fixation within 24 h of admission by less-invasive locked bridge plating, and immediate unrestricted postoperative weight bearing. The primary outcome measure was the rate of postoperative complications. Secondary outcome measures included time to surgery, intraoperative blood loss, duration of surgery, length of hospital stay, time to full weight bearing, and time to radiographic fracture healing.

**Results:**

Fifty four fractures were treated in 52 patients. There were three implant failures, one deep infection, one nonunion and two patients with symptomatic malunion. One patient had knee pain due to patellar component instability associated with valgus alignment. There were ten thromboembolic complications despite consistent anticoagulation. Two patients died within 12 months of injury. Thirty-eight patients had returned to their pre-injury ambulation status at 1 year follow-up.

**Conclusion:**

A standardized approach of less-invasive locked plating fixation and immediate unrestricted weight bearing appears safe and feasible in the management of this vulnerable patient cohort.

**Trial registration number:**

This is a retrospective observational study without a Trial registration number.

## Background

Periprosthetic distal femur fractures around total knee replacements in elderly patients are increasing in incidence and associated with high mortality [1–4]. Limiting weight bearing status after surgery has been associated with a prolonged recovery period and an increased risk of sustaining postoperative complications [4–6]. In analogy to insights from elderly patients with acute hip fractures, early mobilization without restrictions and full weight bearing appears to improve the functional postoperative outcome and decrease mortality [7–9]. As periprosthetic distal femur fractures are by definition extraarticular, restricted weight bearing protocols do not appear justified for this selected cohort of patients and may indeed represent one of the underlying root causes of high complication rates and poor outcomes [10, 11]. The present study was designed to test the hypothesis that a protocol of immediate unrestricted weight bearing after less-invasive locked bridge plating of periprosthetic fractures around the knee is safe and feasible, and not associated with increased rates of implant failure and postoperative complications.

## Methods

A retrospective observational cohort study was performed of all patients managed by locked plate fixation of periprosthetic distal femur fractures with stable prostheses at a Level 1 trauma center during 4-year study time-window from January 1, 2011 to December 31, 2014. Institutional IRB approval was obtained for review of patient charts and medical records. Patients were treated by a standardized institutional protocol which includes admission to a hospitalist service from the emergency department, surgery within 24 h of admission, standardize thromboembolic prophylaxis initiated prior to surgery, minimally invasive locked plating, postoperative weight bearing as tolerated and standardized follow up for 1 year. Pertinent data collection included demographics, time to surgery, intraoperative blood loss, length of surgery, perioperative complications, length of stay, disposition status, time to full weight bearing, time to fracture healing, and postoperative complications including malunion, nonunion, implant failure, and surgical site infections. Data was collected retrospectively from examination of hospital records and the patients chart. Inclusion criteria were all periprosthetic fractures of the distal third of the femur with a stable total knee arthroplasty and surgical management by less-invasive locked plate fixation. Exclusion criteria were high-energy and multiply injured patients with ISS > 15, nonoperative treatment of periprosthetic femur fractures, and patients with an unstable prosthesis requiring revision arthroplasty.

The standardized treatment protocol consisted of initial evaluation in the emergency department with placement of a long posterior splint. A hospitalist team or the trauma service evaluated and admitted the patient primarily depending upon the mechanism of injury and the presence of associated injuries. Venous thromboembolism prophylaxis with low molecular weight heparin (LMWH) was initiated at admission unless contraindicated (e.g., for head injuries, intracranial hemorrhage, preexisting anticoagulation or a known clotting disorder). VTE prophylaxis was not interrupted for surgery unless there was evidence of hemodynamic instability or active bleeding. Patients were optimized for surgery with a goal of completing surgical care within 24 h of admission. All surgeries were performed on a radiolucent table without traction and with the use of intraoperative fluoroscopy. Preoperative antibiotics were administered and continued for 24 h after surgery.

All patients received orders for weight bearing as tolerated and a physical therapy consultation on postoperative day 1. Patients were discharged to a nursing home, rehabilitation center or home depending upon their overall physical capability and social situation. Postoperative visits occurred at 2 weeks, 8 weeks, 14–16 weeks, 6 months and 1 year. At the 2 week visit, staples were removed, therapy was initiated if not already started in the nursing home or rehabilitation center. VTE prophylaxis was managed by the hospitalist team with the collaboration of primary care physicians. Radiographic examination was performed at all outpatient Orthopaedic visits except the 2 week postoperative visit. AP and lateral radiographs were obtained and interpreted by the treating surgeon. Patients with questions regarding alignment received weight bearing radiographs at the 3, 6 and or 12 month visits.

Healing time was based on examination of the office records for each patient. Clinical union was defined prior to the start of the study as pain free weight bearing and absence of pain during stress examination in the office. Radiographic union was defined as 3 of 4 bridging cortices on orthogonal radiographs. Healing was defined as the presence of clinical and radiographic union. Nonunion was defined as the absence of healing at 8 months.

Range of motion was documented at each follow-up up visit based on clinical examination by the treating surgeon. Issues regarding patellar tracking, gait alignment, leg length discrepancy and exercise tolerance were also noted in each case.

Weight bearing status at each follow-up was documented in the medical record. No precise measurements were used to quantify weight bearing. Patients and their families were asked how far they were walking, what types of ambulatory aid they were using, whether they had pain and to what degree their current ambulation ability matched their pre injury mobility. No other outcome parameters were systematically recorded.

Defined complications included superficial or deep wound infection, wound dehiscence without infection, deep vein thrombosis, death or other significant morbidity in the 30 day perioperative period, nonunion, malunion, implant failure and symptomatic knee pain that was not present prior to the injury. Superficial infection was defined as a wound infection not requiring surgery that was treated successfully with local wound care and oral antibiotics. Deep infection was defined as a culture positive infection requiring surgical debridement and cleaning with appropriate antibiotic therapy. Deep vein thrombosis was defined as ultrasound positive evidence of symptomatic clot with subsequent treatment by the medical team. Routine ultrasound screening was not performed on these patients, therefore all patients who received an ultrasound did so due to symptoms of new onset leg or thigh pain and swelling, or suspicion of pulmonary embolus. Implant failure was defined as plate or screw loosening or fracture that was symptomatic or associated with nonunion or malunion. New onset knee pain was defined as knee pain not present prior to surgery and apparently associated with range of motion or ambulation.

### Surgical technique

**Fig. 1:**
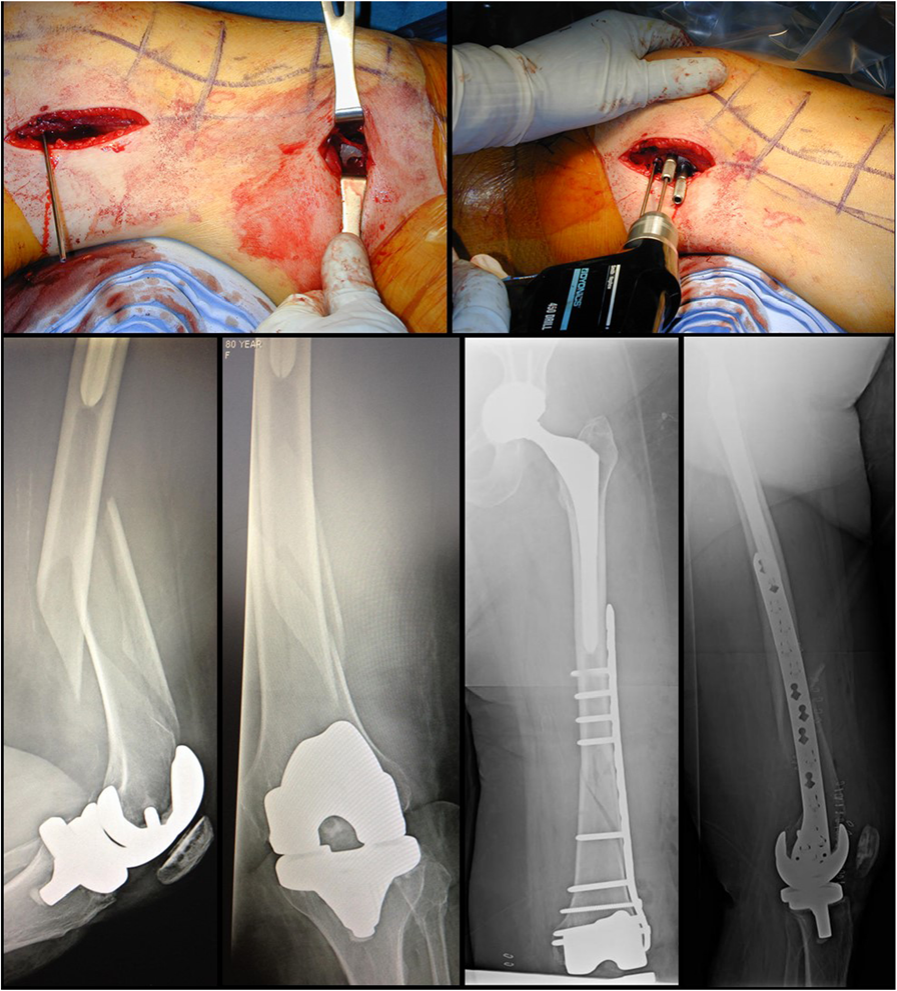
Case example of a periprosthetic left distal femur fracture managed by less-invasive locked plating fixation

The surgical technique included 4–8 cm incisions centered over the lateral distal femur. With a radiolucent triangle or bump, manual traction was performed to align and reduce the fracture. A distal femoral locking plate (Synthes, Stryker, Smith&Nephew) was sized and passed proximally through the lateral incision. Stabilizing wires were placed distal and proximal to anchor the plate through the jigs provided by the particular manufacturer. Various standard fixation techniques were used to obtain an appropriate reduction including the use of “whirlybirds,” conventional cortical screws to pull the bone to the plate and percutaneously placed reduction clamps. The shaft was always affixed to the plate first to establish length and translation. The metaphyseal block was then manipulated into a reduced position relative to the plate to minimize rotational and angular deformities. This maneuver was provisionally stabilized by K-wires through the plate and large circular reduction clamps. Once satisfactory alignment had been achieved based on direct observation and C-arm evaluation, definitive screw placement was performed. In the distal segment, the maximum number of screws possible was placed to decrease the chance for cut-out. In the shaft 4–5 screws were placed, in a bridge plate type construct, with several holes left unfilled to reduce strain at the fracture site. Plate length was dictated by the length of the fracture zone with a goal of 4–5 screws proximally with 3–5 open holes proximally. After staple closure, patients were placed in a knee immobilizer for 2–3 days for pain control and soft tissue management. The knee immobilizer was removed in all cases prior to discharge or transfer. A case example of thesurgical technique is shown in Fig. 1.

## Results

Fifty four fractures were treated in 52 patients. 72% were female. Mean age was 74 (range 52–89). Fifty fractures (93%) healed within 20 weeks (mean 16 weeks). The average range of motion was 3° of extension to 110° flexion. There were three implant failures, one deep infection, one nonunion and two patients with symptomatic malunion (4%). One patient had knee pain due to patellar component instability associated with valgus alignment. There were 9 symptomatic DVTs (17%) and one PE, despite consistent anticoagulation. Two patients died within 12 months of injury (3.7%). Thirty eight (73%) patients by 1 year had returned to their pre injury ambulation status. In two of the three implant failure patients there were identifiable technical errors, notably short plates compared to the fracture length. In both cases, unicortical screws were used in the proximal shaft and these pulled out. In one patient, this presented as new onset pain and nonunion, necessitating revision to a longer implant with bicortical proximal screws. The other case showed pullout and implant failure at follow-up radiographic examination, but the patient had achieved successful healing. The implant was left in place. In the third implant failure patient, the plate broke within 8 weeks through an open screw hole in the bridging zone of the plate. The patient was returned to the operating room for revision with an open reduction with lag screws and new plate and went on to successful healing. The deep infection occurred within 3 weeks of surgery necessitating multiple debridements, placement of antibiotic beads, wound VAC treatment and ultimately union with a healed soft tissue envelope. Implant removal was offered but refused at a later date.

## Discussion

We treated a consecutive cohort of 52 patients with periprosthetic distal femur fractures with a standardized approach including early surgery, minimally invasive locked plating and early mobilization without weight bearing restrictions. We found a low morbidity and mortality rate with this approach. Implant failure occurred in three cases and was attributable to technical errors. There were no nonunions or delayed unions requiring operative intervention. The most common complication was symptomatic deep vein thrombosis despite an aggressive prophylaxis protocol. Overall, a standardized approach which treats these fractures in a manner philosophically similar to hip fractures, with an emphasis on early weight bearing and ambulation, is safe and effective.

Geriatric femur fractures are increasing in incidence [12–14]. Much of the existing literature centers around native hip fracture outcomes regarding morbidity and mortality [15–17]. Distal femur fractures have been shown to have complication rates similar to hip fractures with increasing complication rates and mortality when associated with an existing total hip or knee arthroplasty [1, 4, 18, 19].

Mortality rates for periprosthetic distal femur fractures have been shown to be as high as 17–46% with 30 day, 6 month and 1 year rates of 2%, 13% and 23% respectively [20, 21]. In the present study, our standardized approach of early surgery, less-invasive technique and absence of weight bearing restrictions, we found a reduced mortality rate with one patient death (2.3%) within 12 months of injury. Our protocol emphasizes early mobilization with unrestricted weight bearing. Early mobilization in elderly hip fracture patients has been shown to improve mortality, morbidity and accelerate functional recovery [8, 9, 22, 23]. Limiting weight bearing status after surgery complicates the recovery period by prolonging dependency on walking aids and the need for the patient to remain in an extended care facility [4–7]. By allowing immediate full weight bearing in distal femoral peri-prosthetic fractures we hypothesized this would facilitate functional recovery while maintaining low complication rates. Our study cohort found 31 patients (75%) had returned to their pre injury ambulation status by 1 year.

Historically, weight bearing has been limited post operatively due to concern for high fixation failure rates up to 26% with open reduction [5]. Bridge plate techniques with hybrid locking screw fixation promote an appropriate biomechanical environment facilitating micromotion at the fracture site and union by secondary bone healing [10, 24–29]. Our cohort had a 93% union rate by 20 weeks with one nonunion and two implant failures (4.5%). Our union rate compares favorably to other studies in which weight bearing was delayed (69–89%) [5, 30].

Our protocol employed the use of minimally invasive plate osteosynthesis (MIPO) via submuscular plating techniques (SMP). Complication rates as high as 37% have been reported with open reduction techniques [5]. Indirect reduction and preservation of biology with MIPO/SMP techniques has been shown to have lower complication rates with decreased nonunion risk [30]. Using the MIPO/SMP technique our study cohort had a 93% union rate with low complication rate (one deep infection, two implant failures, one nonunion and two patients with symptomatic malunion). Indirect reduction techniques can increase the risk of malunion. Our study had a symptomatic malunion rate of 4.0% which compares well with reported malunion rates of 13.9% using both open and SMP techniques [30]. The malunion rate in our series was higher than 4.0% overall. However, most patients, despite some element of malunion in at least one plane, were asymptomatic and did not note a functional problem. Trading an increased asymptomatic malunion rate for decreased complications and early weight bearing may be an equitable balance in the geriatric population.

There are several weaknesses in our study design. We did not perform specific outcome measures or use a validated outcome tool to assess the ambulation status or satisfaction of our patients. While such measures are critical in comparison studies between techniques, we felt that the added expense and time was of low yield due to the specifics of our patient population. These patients present acutely with wide variations in pre-injury functional status. A certain percentage have mild to severe cognitive impairments and in many cases their family members are inaccurate historians. Therefore, conclusions based on validated tools, while theoretically more rigorous, may be misleading due to inaccuracy of the input data. We felt the most accurate assessment regarding ambulation outcome, given our specific hypothesis, was based on the follow up history, physical and radiographic examinations. These were all performed by the operative surgeon who was able to integrate the family analysis of function into the medical record for those patients with memory impairment. A second potential weakness is that we used implants from 3 different companies based on surgeon preference. There are no significant differences in the design or technique of these implants and there were no specific failure patterns unique to one implant or another. There was a trend toward pullout failure when only unicortical screws were used. We feel that the outcome of minimally invasive fracture fixation with an appropriate sized locked plate designed for the distal femur is more dependent upon surgeon technique and following sound surgical principles than the type of implant. For those who believe otherwise, a larger volume comparison study would be in order, which was not the purpose of this investigation. Lastly, the strengths of this study include that it was performed at a single center, with a standardized perioperative management protocol and all surgeries were performed by experienced fellowship-trained orthopaedic trauma surgeons who use similar reduction and fixation techniques. All patients were seen by the same surgeons in follow up and treated by the same hospitalist physicians for medical management and DVT prophylaxis. There was 100% patient follow-up during the study period.

## Conclusion

Our findings represent a significant overall improvement compared to historical treatments and are likely due to overall better care in geriatric fracture management as well as technical advances in fracture fixation. We recommend fixating periprosthetic distal femur fractures with minimally invasive locked plating and encouraging immediate weight bearing in every case. Our study provides compelling evidence that determining weight bearing status on a case by case basis is unnecessary and may be deleterious for the patient.

## Abbreviations

DVT: Deep venous thrombosis
IRB: Institutional review board
LMWH: Low molecular weight heparin
MIPO: Minimally invasive plate osteosynthesis
SMP: Submuscular plating
VTE: Venous thromboembolism
